# A Young Healthy Male with Spontaneous Subcutaneous Emphysema Occurring in Neck, Retropharyngeal and Mediastinal Spaces

**DOI:** 10.1155/2020/6963796

**Published:** 2020-02-07

**Authors:** Moayyad Malas, Nawaf Fatani, Zuhair Aljuhani

**Affiliations:** ^1^Department of Otolaryngology, King Abdulaziz Medical City, National Guard Hospital, Jeddah, Saudi Arabia; ^2^Department of Otolaryngology, King Abdulaziz University Hospital, Jeddah, Saudi Arabia; ^3^Department of Otolaryngology, King Fahad General Hospital, Ministry of Health, Jeddah, Saudi Arabia

## Abstract

We present a case of spontaneous cervical, retropharyngeal, and mediastinal emphysema occurring in a previously healthy young male, which is the first described case in Saudi Arabia. The patient was admitted to the ward for observation, monitoring of vital signs, analgesia, and prophylactic antibiotics. The patient was kept under observation for 8 days. During that time, neck pain improved gradually with no episodes of oxygen desaturation or vital sign deterioration. The patient was later discharged with very mild persistent pain. Two weeks after discharge, patient was seen in outpatient clinic and was free of symptoms. Spontaneous subcutaneous emphysema remains a rare presentation encountered in emergency department. The investigations and treatment required for such patients has no consensus between authors. Although most of the reported cases described an uncomplicated course, there is a need for clear guidelines on management protocol.

## 1. Introduction

Emphysema is described in Greek as a swelling or inflation. It is used usually in medical terminology to describe chronic lung disease where air is trapped inside the lungs. This term, however, is also used to describe a collection of air in a limited space in the body, as in cervical, subcutaneous, or mediastinal emphysema. Subcutaneous emphysema is reported in the literature as primary or secondary to other etiologies, such as surgery [1], dental procedures [2, 3], trauma, lung disease [4], or infectious process [5]. The incidence of spontaneous emphysema occurring in the mediastinum [6, 7], cervical [8], or retropharyngeal [9] was mentioned previously in the literature. However, the occurrence of emphysema spontaneously and involving all three mentioned spaces simultaneously was rarely described in the literature [10].

## 2. Case Presentation

We present a case of a 21-year-old male who presented to the emergency department complaining of neck pain. The pain had started the night before presentation, a few hours after lifting heavy objects. The pain was in the anterior neck and upper chest, stabbing in nature, continuous, increases when moving the head, lying flat, and with deep breathing. The patient had no history of trauma, asthma, odynophagia, or dysphagia. He does not have any comorbidities or previous surgeries. Patient mentioned history of occasional hookah smoking.

On examination the patient was vitally stable. He looked well, sitting comfortably on emergency room bed without shortness of breath, or apparent facial or neck swelling. Oxygen saturation was maintained at 99% on room air. Neck examination showed central trachea, tenderness in anterior neck, and crepitation under the skin. No palpable masses, lymph nodes, or thyroid tissue. Endoscopy was performed to the nasal cavity, nasopharynx, oropharynx, and larynx, which showed no airway narrowing or edema. Chest and cardiovascular examination was unremarkable except for tenderness in the anterior chest wall.

**Figure 1:**
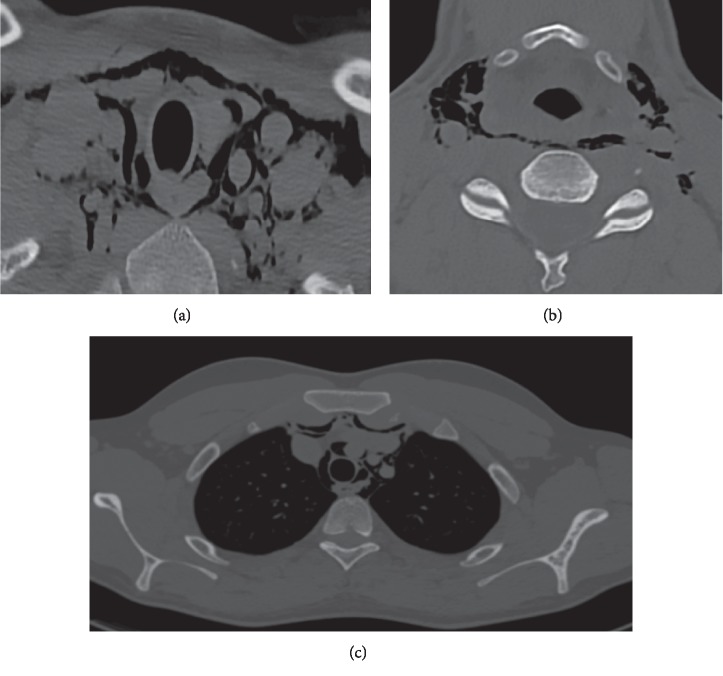
Contrast enhanced CT scan axial images for patients with spontaneous subcutaneous emphysema of the neck and anterior medistinum. (a) Emphysema occurring in neck spaces: parapharyngeal, carotid, and pretracheal spaces. (b) Emphysema in retropharyngeal space with parapharyngeal extension. (c) Emphysema also extending to mediastinal space and some air pockets are seen retrosternal.

Blood investigations, including complete blood count and renal and liver profiles, showed results within normal limits. Computed tomography (CT) scan of the neck and chest with contrast showed diffuse subcutaneous, soft tissue, and intermuscular head and neck emphysema surrounding the visceral, carotid, retropharyngeal, and posterior cervical spaces reaching down to the anterior mediastinum causing mild pneumomediastinum. There is no pneumothorax, tracheal, laryngeal, or esophageal injury, as shown in (Figure 1).

**Figure 2:**
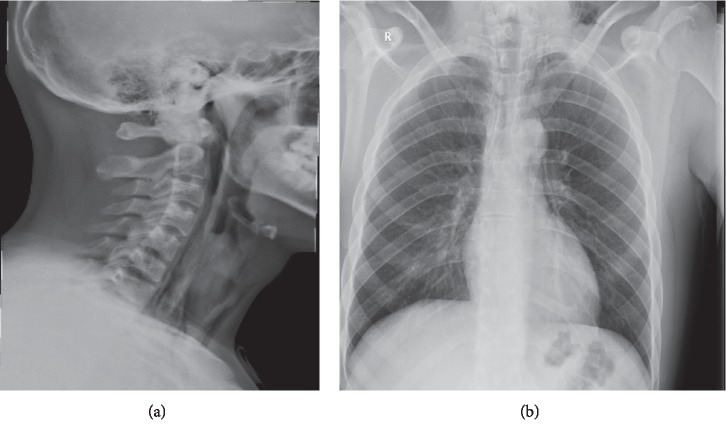
Lateral neck (a) and posteroanterior chest (b) X-rays at time of presentation showing air in neck spaces and chest has minimal air around the trachea but no pneumothorax seen and lung fields are clear.

**Figure 3:**
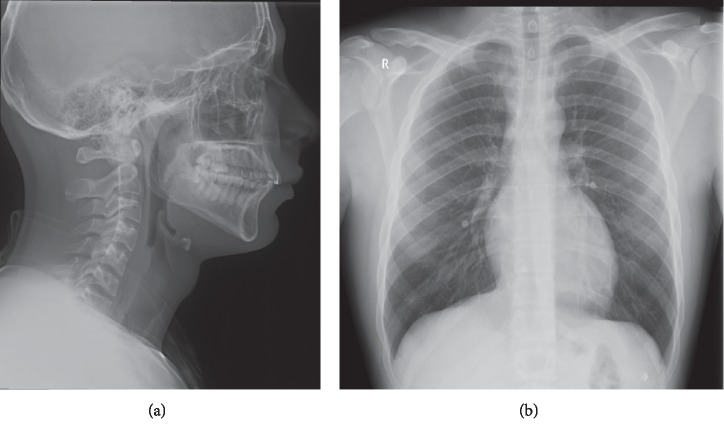
Lateral neck (a) and posteroanterior chest (b) X-rays after 2 weeks showing resolution of previously shown air in the neck and anterior mediastinum.

The patient was admitted to the ward for observation, monitoring of vital signs, analgesia, and prophylactic antibiotics. The patient was kept under observation for 8 days. During that time, neck pain improved gradually with no episodes of oxygen desaturation or vital signs deterioration. The patient was later discharged with very mild persistent pain. Two weeks after discharge, the patient was seen in outpatient clinic and was free of symptoms. Lateral neck and posteroanterior chest X-rays at presentation and 2 weeks later are shown in Figures 2 and 3.

## 3. Discussion

Subcutaneous emphysema was described in the literature beginning in the late 1910s by Berkeley and Coffen [11]; then, Meyer and Lucke [12] described another case in 1920. Later, Bloomberg also reported one of the speculated mechanisms of air leak to cause pneumomediastinum and cervical emphysema [13]. Although there are multiple etiologies listed in the literature, the occurrence of spontaneous subcutaneous emphysema is uncommonly described, especially when it occurs concurrently in the mediastinum, cervical, and retropharyngeal spaces.

The causes of subcutaneous emphysema can be classified into idiopathic and secondary. Some authors also classified it as tuberculous and nontuberculous etiologies [13]. Many of the case reports available in the literature describe secondary subcutaneous emphysema [1, 5, 14, 15], and, to our knowledge, only a few describe idiopathic subcutaneous emphysema [9, 16].

In Saudi Arabia, the incidence of secondary subcutaneous emphysema was reported in two studies; the first was reported by Abo Essa et al. where 2 cases of subcutaneous emphysema and pneumomediastinum in children with H1N1 [14] were reported. Another report by Abdullah et al. described a rare presentation of a patient with pneumomediastinum caused by duodenal ulcer [17]. Nevertheless, no cases of spontaneous mediastinal, cervical, and retropharyngeal subcutaneous emphysema were described.

The course of spontaneous cervical emphysema is usually benign. With the use of conservative treatment including analgesia, most of the patients' symptoms gradually resolve without requiring any further intervention or treatment. Some authors, however, suggest the use of prophylactic antibiotics [9], while others recommend only pain control and treatment of other illness, if present [8, 18, 19]. In our case, we kept the patient for observation for 8 days on prophylactic antibiotics. He was then discharged home on analgesics. The patient was later followed up in the outpatient clinic where he was found to be asymptomatic.

## 4. Conclusion

Spontaneous subcutaneous emphysema remains a rare presentation encountered in emergency department. The investigations and treatment required for such patients has no consensus between authors. Although most of the reported cases described an uncomplicated course, there is a need for clear guidelines on management protocol.
